# Supplementary material for: Comparative multiomics analysis of cell physiological state after culture in a basket bioreactor
Source: Sci Rep. 2022 Nov 23;12:20161. doi: 10.1038/s41598-022-24687-4 (PMC9686226; doi:10.1038/s41598-022-24687-4)
Supplement: Supplementary file 1 — Supplementary Information 1. [file 41598_2022_24687_MOESM1_ESM.zip › raw data/Transcriptomic raw data/6.Enrichment/2.KEGG/TE1vsTC1/UP/TE1vsTC1.up_KEGGdot.pdf]

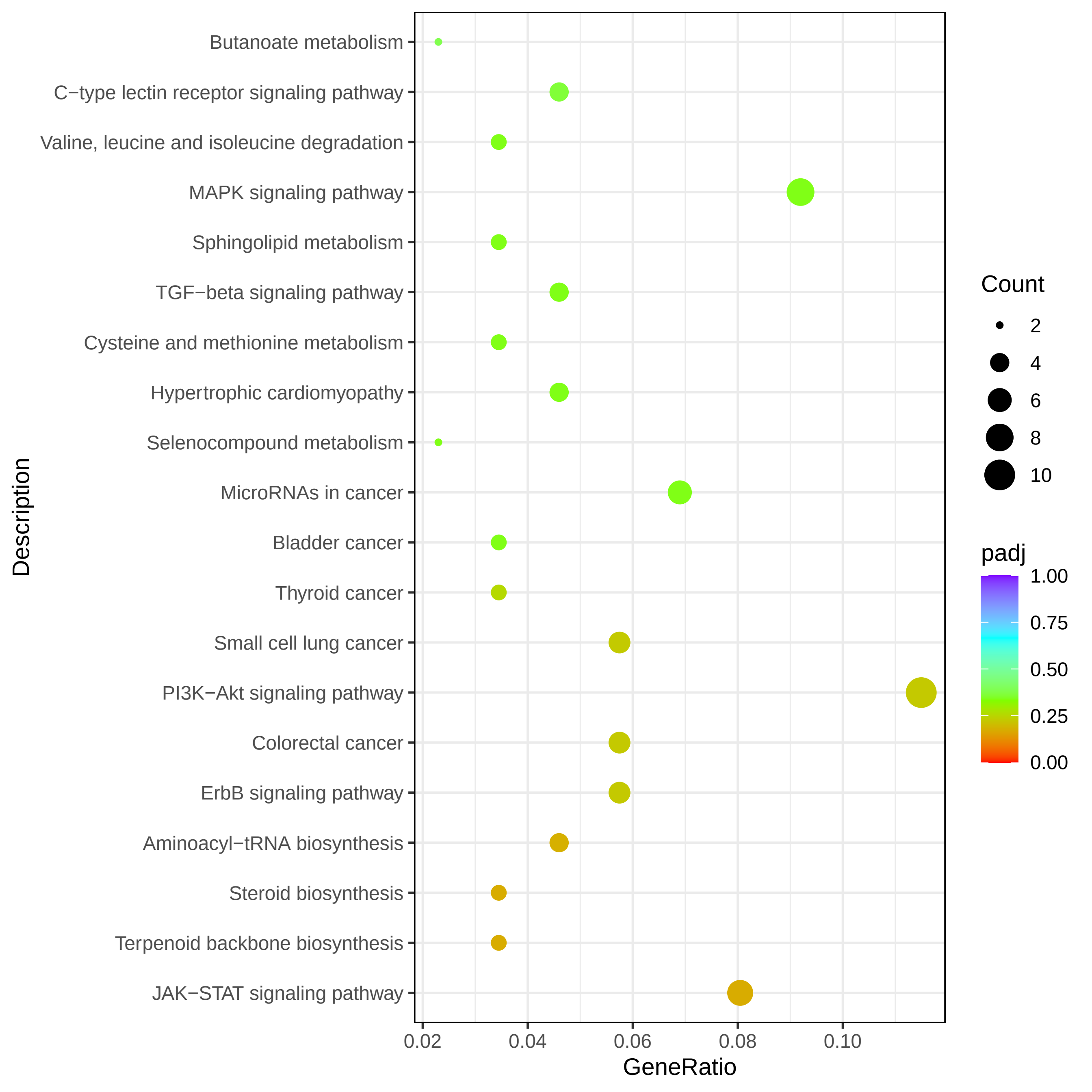

Butanoate metabolism
C−type lectin receptor signaling pathway
Valine, leucine and isoleucine degradation
MAPK signaling pathway
Sphingolipid metabolism
TGF−beta signaling pathway
Cysteine and methionine metabolism
Hypertrophic cardiomyopathy
Selenocompound metabolism
MicroRNAs in cancer
Bladder cancer
Thyroid cancer
Small cell lung cancer
PI3K−Akt signaling pathway
Colorectal cancer
ErbB signaling pathway
Aminoacyl−tRNA biosynthesis
Steroid biosynthesis
Terpenoid backbone biosynthesis
JAK−STAT signaling pathway
Description
0.02
0.04
0.06
0.08
0.10
GeneRatio
Count
2
4
6
8
10
padj
1.00
0.75
0.50
0.25
0.00
